# Explaining differences in education-related inequalities in health between urban and rural areas in Mongolia

**DOI:** 10.1186/s12939-015-0281-9

**Published:** 2015-12-22

**Authors:** Javkhlanbayar Dorjdagva, Enkhjargal Batbaatar, Bayarsaikhan Dorjsuren, Jussi Kauhanen

**Affiliations:** Department of Health Policy and Management, School of Public Health, Mongolian National University of Medical Sciences, Ulaanbaatar, Mongolia; Institute of Public Health and Clinical Nutrition, Faculty of Health Sciences, University of Eastern Finland, Kuopio, Finland; Faculty of Economics and Business Sciences, University of Sannio, Sannio, Italy; Department of Health Systems Governance and Financing, World Health Organization, Geneva, Switzerland

**Keywords:** Health inequality, Equity, Decomposition analysis, Urban and rural disparity, Mongolia

## Abstract

**Background:**

After the socioeconomic transition in 1990, Mongolia has been experiencing demographic and epidemiologic transitions; however, there is lack of evidence on socioeconomic-related inequality in health across the country. The aim of this paper is to evaluate the education-related inequalities in adult population health in urban and rural areas of Mongolia in 2007/2008.

**Methods:**

This paper used a nationwide cross-sectional data, the Household Socio-Economic Survey 2007/2008, collected by the National Statistical Office. We employed the Erreygers’ concentration index to assess the degree of education-related inequality in adult health in urban and rural areas.

**Results:**

Our results suggest that a lower education level was associated with poor self-reported health. The concentration indices of physical limitation and chronic disease were significantly less than zero in both areas. On the other hand, ill-health was concentrated among the less educated groups.

The decomposition results show education, economic activity status and income were the main contributors to education-related inequalities in physical limitation and chronic disease removing age-sex related contributions.

**Conclusions:**

Improving accessibility and quality of education, especially for the lower socioeconomic groups may reduce socioeconomic-related inequality in health in both rural and urban areas of Mongolia.

## Background

The association between socioeconomic status (SES) and health has been extensively documented in the international literature. Current evidence suggests lower SES indicates such being poor, less educated or unemployed are generally associated with a higher risk of mortality, ill-health, unhealthy behaviors and a lack of health care access in both the developing and developed world [1].

Tackling avoidable inequalities in health and improving population health among lower SES groups health have been a central issue for health policy analysis and studies, especially in low and middle income countries [2], including Mongolia [3].

Mongolia developed under socialist ideology for many decades until the country started market oriented economic reform in 1991. During 70 years of socialism, substantial attention was paid to education and public health in Mongolia. As a result, average life expectancy was raised from 46.7 in 1960 to 63.7 with universally accessible free health care services to everyone by 1990 [4]. In addition, during the 1970s and 1980s, the country’s education system was observed to be as one of the best in the developing world, and the adult literacy rate reached 97.8% in 1990 [4, 5]. In 1990, there was a peaceful democratic revolution, a transition from a centrally planned economy to a market economy. During the transition, the country experienced an economic crises and collapse starting from 1992, which led to rapid and fundamental reforms in the public service sectors, including the health sector [6]. Consequently, social health insurance was introduced in 1994 [7], and its coverage reached 98% in 2011 [6]. Furthermore, public-private-partnership was initiated to increase the role of private sector in provision of health care services and products such as medicine. The reform also supported administrative decentralisation and management of social services, but primary health care remained a key priority for the central and local governments to maintain and improve health sector performances during transitional period. Today, everyone has free access to primary health care, which is fully funded by the state budget [6].

Along with rapid socioeconomic changes, over the last two decades, Mongolia has been facing both demographic and epidemiologic transitions. Mongolia, with a population of 3 million [8], is the 19^th^ largest country in the world. Despite being one of the most sparsely populated countries, in 2013, 68% of the total population were living in urban areas, with 46.8% of the total population in the capital city itself; this could be due to the internal migration flow during the past two decades [9].

In addition to the demographic transition, the segment of population 60 and older is projected to grow from 6 to 25% by 2050 [10]. In terms of the epidemiologic transition, the leading causes of death have shifted from communicable diseases to preventable and lifestyle-related diseases, such as cardiovascular diseases, cancers, and injuries, since 1990 [11]. During the same period, maternal and child mortality rates have decreased; however, the adult mortality rate has been on the rise [6].

Available data, information, evidence and literature, suggest that SES and health are associated in many ways in Mongolia. For instance, an earlier study concluded that lower education level or unemployment was associated with poor self-reported health in Mongolia [12]. Furthermore, mothers with no education were at a higher risk of having babies with low birth weight [13]. In terms of geographical differences, more than 40% of the maternal mortality was observed among rural female herders [6]. However, the majority of the previous studies were descriptive in design.

In our previous study, we analyzed income-related inequalities in health care utilization and changes in them between 2007/2008 and 2012 in Mongolia employing Erreyger’s concentration index [14]. We found income-related inequalities in health care utilization tended to increase over time. Specifically, health care services at the tertiary level as well as private hospitals were pro-rich, and health care services in lower-level hospitals and family group practices were pro-poor. Affordability, location, and education were the major contributors to socio-economic related inequality in health care utilization, particularly for the low income group. To our judgement, the previous study results in Mongolia are still insufficient to make assumptions that there is persistent socioeconomic-related inequality in health. There is only one study that addressed socioeconomic-related inequality in health in Mongolia. However, that study used merely child health indicators as health outcome variables and found out that ill-health indicators, such as stunting, underweight, and diarrhea, was concentrated among the lower socioeconomic group [15]; it could not depict a broader picture of health inequality in Mongolia. In addition the country has been facing demographic and epidemiologic transitions after the socioeconomic changes. In this light, measuring socioeconomic-related inequality in adult population health and identifying its main contributing factors are policy relevant essential issues for the country.

This paper provides new evidence on socioeconomic-related inequality in health in Mongolia. The SES can be measured by education, income or occupation. In this study we use education attainment as a living standard variable.

Firstly, we estimate education-related inequality in health based on adult health variables; we use self-reported health as indicator. Secondly, we compare urban and rural differences in the degree of health inequality. Understanding the health distribution of urban and rural areas is crucial since the country is experiencing drastic urbanization. Finally, we employed the Erreygers’ concentration index (EI), which is more compatible with binary health outcome variables [16].

The aim of this paper is to evaluate education-related inequalities in adult population health in urban and rural areas of Mongolia in 2007/2008.

## Methods

### Data

In this paper, we used a nationwide cross-sectional data, the Mongolian Household Socio-Economic Survey (HSES) 2007/2008, with permission from the National Statistical Office. The HSES 2007/2008 was designed to “evaluate and monitor the income and expenditure of households, to provide the basis for the poverty monitoring system, poverty mapping and poverty reduction policies, to update the basket and the weights for the consumer price index, and to offer inputs to the national accounts”. The survey consisted of questions regarding demographics, socio-economic indicators, social transfers, household income and expenditure, housing and education, inter alia. The third section of the questionnaire addressed individuals’ health status, health care utilization as well as health expenditure. The survey included 11,172 households with 44,510 individuals. The main inclusion criterion was that respondents be 18 or older. We excluded respondents who were: a) a household head or any household student members away from home for the past 11 months or more; b) anyone else away from home for the last 6 months or longer. After applying the inclusion criteria, cases with missing data were eliminated, a set of 27,666.

### Measuring health status

The third section of the HSES questionnaire addressed respondents’ health status. We used physical limitation and chronic disease as health variables. Both were self-assessed, and thus the measurements were subjective. The respondents were asked: a) do you have any chronic illness?; b) have you got any physical disability?.

Self-assessed health is a convenient outcome measurement. It has been observed that people who rated their health as poor more likely to suffer from subsequent mortality [17, 18]. Therefore, inequality in self-assessed health is a strong predictor of inequality in mortality [19].

### Measuring socio-economic status

Since the concentration index is rank-dependent method of socioeconomic-related inequality in health, a measurement choice of SES is important. A literature stated that the concentration index is sensitive to a type of measurement of SES [20], as outcome varying as a type of SES measurement used.

In our previous study, with a purpose of measuring inequality in health care utilization, income was used as a SES measurement [14]. Although, when it comes to the comparison of inequalities in health between rural and urban areas, income may not be an appropriate measure of SES. Because 27% of the total population are herders and majority of them live in rural areas [9], where the main living source is farming and self-production to support their lives is a common practice. Therefore, real incomes cannot be easily calculated. In this sense, we chose education as a measure of socio-economic inequalities in health owing to its indistinguishable higher access in both areas [5].

Individuals’ educational attainment levels in the survey questionnaire were divided into eight levels. We, however, recoded them into four in order to be consistent with the International Standard Classification of Education (ISCED). None or low education is correspondent to ISCED 0 to 1, lower secondary to ISCED 2, upper secondary to ISCED 3 to 4, and tertiary education to ISCED 5 to 6.

### Independent variables

We created 14 dummy variables regarding age and gender (females aged 18–24, 25–34, 35–44, 45–54, 55–64, 65–74, and 75 or older; males aged 18–24, 25–34, 35–44, 45–54, 55–64, 65–74, and 75 or older). The reference group was females, aged 18 to 24. The four types of marital status were included married/living together (baseline), divorced/separated, widowed and single/never married. Economic activity status was measured by categories, employed (the reference group), herder, self-employed, inactive (student, retired, doing housework and other economically inactive groups), and unemployed. Household size was a continuous variable. We measured household income based on amount earned by any household member during the study years from any type of the following: wage from work, income from self-employment, agricultural income, private income and pension, among others. In accordance with the OECD modified equivalence scale (giving a weight 1 to the household head, 0.5 to each additional adult member and 0.3 to each child), household income per equivalent adult was estimated.

### Measuring inequality

We used the concentration index to measure socioeconomic-related inequality in health, which is the most commonly used method in this field [21]. Since our ranking variable was education, education-related inequality in health will be the term we use now on; this inequality is, certainly, a part of the total inequality.

The concentration index is directly linked to the concentration curve, which enables us to see the whole picture of a share of health by cumulative proportions of population ranked by socioeconomic status [22]. Thus, in our case, the concentration index indicates the covariance of the health and the fractional rank of education attainment distribution as

1 $$ CI = \frac{2}{\mu }\ co{v}_w\left({h}_i,\ {R}_i\right) $$

where *i* is an individual, *h*_*i*_ is the health/ill-health variable, *μ* is the mean of the health variable (*h*), *R*_*i*_ is the fractional education rank of individual *i*. The margin of the concentration index is between -1 and +1. A negative value of the concentration index indicates ill-health (in our case, any chronic disease or physical limitation) is concentrated among the poor. In contrast, a positive value indicates that ill health is higher among the rich. A zero index shows there is no socioeconomic-related inequality in health.

Nonetheless, the concentration index has a limitation. When a health variable is binary, as the mean increases, the concentration index shrinks [23]. There are some corrections for the concentration index suggested in practice [16, 23]. Among them, EI’s and Wagstaff’s concentration indices are the most commonly used, and the choice is based on researcher preference. In this paper we use EI because EI is the only indicator in the family of techniques which satisfies the requirements of four key properties of the rank-dependent indices [24].^1^ It is formulated as thus:

2 $$ E(h) = \frac{4\upmu}{\left({b}_n-{a}_n\right)}\ C(h) $$

where *C(h)* represents the standard concentration index, which is presented in Eq. 1. The *μ* is the mean of a health variable in population. *b*_*n*_ and *a*_*n*_ are the upper and lower bound of the health variable.

### Demographic standardization

Health inequality is categorized as potentially avoidable and unavoidable health inequality. Apparently, the distribution of health differs among and across the populations as regards the education differences because health status varies in the population due to individuals’ demographic and background differences, such as age and gender. These differences arise naturally and unavoidable. Therefore, in order to assess whether health is equally distributed in the population regarding education distribution, one should control varying demographic variables. In other words, potentially avoidable inequality or inequity in health is expressed by a difference of actual health inequality in the population and age-sex standardized health inequality [21].

We used ordinary least squares (OLS) regression in both indirect standardization and decomposition analysis even though our outcome variables are binary. When studies used both non-linear estimation and OLS to make comparisons, the results were consistent in both the linear and non-linear models [22, 25]. Additionally, Van Doorslear and others observed that the decomposition analysis introduces an approximation error in non-linear models [26].

Thus, first, coefficients of the OLS for ill-health, including physical limitation and chronic disease (*h*_*i*_) were estimated as following:

3 $$ {h}_{i}= \alpha + \sum\limits_{j}{\beta}_{j}{\chi}_{j,i}+\sum\limits_{k}{\gamma}_{k}{Z}_{k,i}+{\varepsilon}_{i} $$

where *h*_*i*_ is the health variable, *i* represents the individual; *χ*_*j*_ are demographic variables (e.g., age and sex); *z*_*k*_ are non-demographic variables consisting of education, logarithm of household income, marital status, economic activity status, household size; and *α*,*β* and *γ* are the parameter vectors, and *ε*_*i*_ is an error term.

Second, we generated predicted values of health (*ĥ*_*i*_^*x*^) using the parameter vectors (*α,β*_*j*_*,γ*_*k*_), individual values of demographic variables (*χ*_*j*_), and sample means of the non-demographic (*z*_*k*,*i*_) variables from Eq. 3. The predicted values were obtained by the formula below:

4 $$ {\widehat{h}}_i^x=\widehat{\alpha} + {\displaystyle \sum_j}{\widehat{\beta}}_j{\chi}_{j,i} + {\displaystyle \sum_k}{\widehat{\gamma}}_k{z}_k^m $$

Finally, the estimate of indirectly standardized health (*ĥ*_*i*_^*IS*^) was simply obtained from the difference between actual (*h*_*i*_) and need-predicted health (*ĥ*_*i*_^*X*^), and the sample mean (*h*^*m*^) was added [22].

5 $$ {\widehat{h}}_i^{IS} = {h}_i - {\widehat{h}}_i^X + {h}^m $$

### Decomposition analysis

By using decomposition analysis, we can observe which determinant contributes independently more to education-related inequality in health [22]. As previously mentioned, the most convenient method to decompose is the linear additive model [26]. The EI is employed due to nature of binary health variables instead of the standard concentration index, and the decomposition of concentration index was multiplied by 4 and *μ*_h_ to obtain EI (Eq. 6).

6 $$ E=4\left[{\displaystyle \sum_j}{\beta}_j{\mu}_{x_j}{C}_{x_j}+{\displaystyle \sum_k}{\gamma}_k{\mu}_{z_k}{C}_{z_k}\right] $$

where *μ* represents the mean, *β* and *γ* represent the coefficient of the variable *x* and *z*, respectively. C represents the concentration index [16].

We performed statistical analysis using the STATA MP 12.1 (StataCorp LP, TEXAS).

## Results

The results of descriptive statistics for the rural and urban areas are presented in Table 1. It shows that the difference in the mean of adults with physical limitation in urban and rural areas was very slight. There is clear indication that adults living in rural areas suffer more with chronic diseases compared to adults living in urban settlements. Although there were insignificant demographic structure differences between urban and rural areas. Respondents in urban areas reported higher incomes.

**Table 1:** Descriptive statistics

Variable	Urban *(n* = 15,996)	Rural (*n* = 11,670)
	Percent	
Health variables		
Physical limitation	1.8 %	2.0 %
Chronic disease^a^	16.4 %	19.7 %
Age and sex		
Female 18–24^b^	12.2 %	11.6 %
Female 25–34	12.7 %	12.7 %
Female 35–44	12.4 %	11.9 %
Female 45–54^a^	9.1 %	7.6 %
Female 55–64^a^	4.3 %	3.7 %
Female 65–74	2.6 %	2.5 %
Female 74<	1.5 %	1.3 %
Male 18–24	11.1 %	11.3 %
Male 25–34^a^	10.5 %	12.8 %
Male 35–44^a^	10.0 %	11.2 %
Male 45–54	7.5 %	7.3 %
Male 55–64^a^	3.4 %	3.0 %
Male 65–74	2.0 %	2.3 %
Male 74<	0.8 %	0.8 %
Log income per capita. median. min and max a	14.2 (9.2, 19.4)	13.7 (6.9, 18.6)
Marital status		
Married/living together ^a,b^	57.8 %	63.7 %
Divorced/separated	4.8 %	1.7 %
Widowed	8.9 %	8.1 %
Single/never married	28.5 %	26.6 %
Employment status		
Employed^a,b^	38.7 %	16.8 %
Herder^a^	1.7 %	29.4 %
Self-employed^a^	14.2 %	30.6 %
Inactive^a^	26.4 %	13.5 %
Unemployed^a^	18.9 %	9.6 %
Education level		
Non or lower education^a,b^	6.6 %	26.3 %
Lower secondary^a^	13.7 %	28.2 %
Upper secondary^a^	42.4 %	29.8 %
Third-level education^a^	37.3 %	15.8 %
Household size. median. min and max^a^	4 (1, 17)	4 (1, 16)

^a^Statistically significant difference (*p* < 0.05) between rural and urban areas

^b^Reference group

**Fig. 1:**
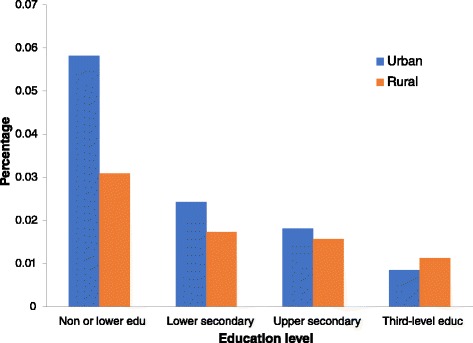
Standardized physical limitation for urban population and rural population by education levels in 2007/2008

**Fig. 2:**
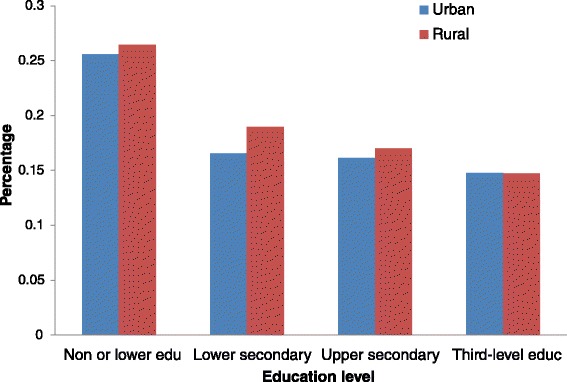
Standardized chronic disease for urban population and rural population by education levels in 2007/2008

In terms of the economic activity status variable, there were higher percentages of employed and inactive groups and lower percentage of the self-employed group in urban areas than in rural ones. Rural areas reported a higher percentage of those married and a lower percentage of the divorced people. Notably, the urban population tended to have significantly better education. For instance, 37% of people in urban areas reported having tertiary education, whereas only 16% had this level in rural areas. Standardized physical limitations and chronic diseases for both urban and rural populations based education levels in 2007/2008 are presented in Figs. 1 and 2. People educated more highly reported fewer chronic diseases and limitations.

The results of regression analysis are presented in Table 2. Reference categories including females aged 18 to 24, married/living together, employed, and none or lower education were omitted.

**Table 2:** OLS results for health variables

	Physical limitation		Chronic disease	
	Urban	Rural	Urban	Rural
Female 25–34	0,0349***	0,0250***	0,0620***	0,0731***
Female 35–44	0,0482***	0,0423***	0,1442***	0,2051***
Female 45–54	0,0512***	0,0447***	0,2483***	0,3150***
Female 55–64	0,0129*	0,0057	0,3677***	0,3977***
Female 65–74	−0,0006	0,0214**	0,4542***	0,4350***
Female 74<	0,0088	0,0062	0,4126***	0,4495***
Male 18–24	0,0047	0,0086	0,0014	0,0269*
Male 25–34	0,0469***	0,0361***	0,0789***	0,0651***
Male 35–44	0,0585***	0,0483***	0,1394***	0,1489***
Male 45–54	0,0701***	0,0590***	0,2141***	0,2599***
Male 55–64	0,0538***	0,0584***	0,3141***	0,3444***
Male 65–74	0,0089	0,0350***	0,4185***	0,4044***
Male 74<	0,0177	0,0190	0,5108***	0,3244***
Log income per capita	−0,0007	0,0045***	−0,0064**	0,0063
Divorced/separated	0,0008	0,0210**	0,0230*	−0,0008
Widowed	0,0016	−0,0057	−0,0015	0,0275*
Single/never married	0,0113***	0,0194***	0,0182**	0,0072
Herder	0,0160*	0,0013	0,0684***	0,0249**
Self-employed	0,0020	0,0114***	0,0077	0,0401***
Inactive	0,0574***	0,0483***	0,0778***	0,0810***
Unemployed	0,0213***	0,0283***	0,0607***	0,1007***
Lower secondary	−0,0139**	−0,0100***	−0,0656***	−0,0620***
Upper secondary	−0,0200***	−0,0184***	−0,0673***	−0,0864***
Third-level education	−0,0190***	−0,0196***	−0,0641***	−0,0988***
Household size	−0,0014**	−0,0020***	−0,0057***	−0,0143***
Constant	−0,0049	−0,0699***	0,1569***	0,0290
N	15996	11670	15996	11670
Prob > F	0	0	0	0
R-squared	0,0303	0,0228	0,1542	0,1566
Adj R-squared	0,0288	0,0207	0,1528	0,1548

p < 0.01***, p < 0.05**, p < 0.1*

As we predicted, physical limitation was reported more with increasing age in both urban and rural areas. However, we observed that males aged 55 to 64 were less likely to report physical limitations than males aged of 45 to 54 in urban areas. In addition, respondents who were married or living together in both rural and urban areas reported lower physical limitations than respondents who had a different marital status. We found that economically inactive respondents tended to report the most physical limitations in both areas. As expected, education attainment was negatively associated with self-reported physical limitation in urban and rural areas. We observed a negative relationship between physical limitation and household size in both areas.

It is worth mentioning that (the logarithm of) household income was positively associated with reporting physical limitations in rural area.

We found a positive association between age and chronic disease with the exception of males aged 75 and older in rural areas and females aged 75 and older in urban areas. Those married or living together were less likely to report chronic diseases in both areas. Individuals who had a job tended to report less chronic diseases than other economic groups. A negative association between education attainment and chronic disease was observed in both areas; however, the impact was higher in the rural population. Household size was negatively associated with chronic disease in both areas. Household income (the logarithm of) was significantly negatively associated with chronic disease in urban areas. The OLS regression results showed that R-squared for chronic disease in urban and rural areas were greater than R-squared for physical limitation in either areas. There were not huge differences between urban and rural areas for both indicators.

### Education-related inequality in physical limitations and chronic diseases

We estimated the concentration indices using Eq. 2. The summary of the results are presented in Table 3, and it contains interesting findings. First, all the EIs were less than zero (*p* < 0.001), indicating that physical limitation and chronic disease were unequally distributed in favour of the lower socioeconomic groups in rural and urban areas. These results suggest that the existence of education-related inequalities in health. Second, the EIs of physical limitation in urban areas was -0.0136 and that of rural areas -0.0131. There were no considerable differences between the areas. Third, in terms of chronic disease, rural areas had much greater education-related inequality (-0.1397) than urban areas (-0.0675). Fourth, chronic disease inequality was substantially greater than physical limitation inequality in both areas.

**Table 3:** Erreyger’s concentration indices of physical limitation and chronic disease

	Physical limitation		Chronic disease	
	Urban	Rural	Urban	Rural
EI	−0,0136	−0,0131	−0,0675	−0,1397
Se	0,0027	0,0031	0,0084	0,0111
I* = EI-C*	−0,0192	−0,0133	−0,0343	−0,0831
*P* value	0,00	0,00	0,00	0,00

I*- Avoidable health inequality

C*- Health inequality due to demographics

In Table 3, I* represents non-demographic inequality or ‘potentially avoidable inequality’. We computed I* by subtracting health inequality due to demographics (C*) from the total inequality. Table 3 shows that *EI* is less than I* for physical limitation in both areas whereas *EI* is greater than I* for chronic disease in both areas. It can be explained as the health and SES distributions of age and sex may have both negative and positive impacts on the total inequality.

In both areas, potentially avoidable inequality, I* was, statistically significant, and less than zero. In terms of chronic disease, the degree of avoidable inequality was higher in rural areas than urban ones. However, degrees of inequality in physical limitation in urban areas was -0.019 and -0.013 in rural areas.

### Decomposition analysis

According to our findings, it is evident that education-related inequality in health existed in Mongolia during the study period. Amount each variable contributed to total health inequality is, however, unclear. Hence, we used Eq. 6 in order to decompose education-related inequality in health. Table 4 shows the results of the decomposition analysis. The second and third columns of Table 4 exhibit concentration indices for determinants by rural and urban areas. There was a concentration of older males and females in the less education group in both areas. As expected, income was concentrated among the highly educated group in both areas. There was a concertation of divorcee among the better educated group in rural and urban areas. Herders, the self-employed and those who are economically inactive were concentrated among the less educated group in both areas, and the same is true for the unemployed but only in urban areas. Big families were concentrated among the less educated groups in urban areas, big families were in the better educated groups in rural areas.

**Table 4:** Decomposition of concentration index for physical limitation and chronic disease in urban and rural areas, Mongolia

	Concentration index		Physical limitation				Chronic disease			
	Urban	Rural	Urban		Rural		Urban		Rural	
			Elasticities	Contribution	Elasticities	Contribution	Elasticities	Contribution	Elasticities	Contribution
Female 25–34	0.1975	0.0881	0.2410	−26.7 %	0.1604	−8.5 %	0.0493	−9.5 %	0.0468	−2.3 %
Female 35–44	0.0864	0.2043	0.3123	−15.1 %	0.2551	−31.4 %	0.1078	−9.1 %	0.1234	−14.3 %
Female 45–54	0.0388	−0.0019	0.2393	−5.2 %	0.1731	0.2 %	0.1339	−5.1 %	0.1215	0.1 %
Female 55–64	−0.0964	−0.2952	0.0302	1.6 %	0.0107	1.9 %	0.0989	9.3 %	0.0749	12.6 %
Female 65–74	−0.4330	−0.5493	−0.0009	−0.2 %	0.0263	8.7 %	0.0753	31.7 %	0.0535	16.7 %
Female 74<	−0.7018	−0.6348	0.0074	2.9 %	0.0042	1.6 %	0.0403	27.5 %	0.0302	10.9 %
Male 18–24	−0.1031	0.0064	0.0269	1.6 %	0.0491	−0.2 %	0.0009	0.1 %	0.0152	−0.1 %
Male 25–34	0.0166	−0.1155	0.2685	−2.5 %	0.2346	16.3 %	0.0521	−0.8 %	0.0421	2.8 %
Male 35–44	0.0011	0.0763	0.3042	−0.2 %	0.2717	−12.5 %	0.0836	−0.1 %	0.0836	−3.6 %
Male 45–54	−0.0157	−0.0434	0.2691	2.4 %	0.2194	5.7 %	0.0947	1.4 %	0.0964	2.4 %
Male 55–64	0.0260	0.1390	0.0961	−1.4 %	0.0884	7.4 %	0.0647	−1.6 %	0.0520	4.1 %
Male 65–74	−0.0037	−0.2958	0.0102	0.0 %	0.0402	7.2 %	0.0553	0.2 %	0.0462	7.8 %
Male 74<	−0.1993	−0.5087	0.0079	0.9 %	0.0072	2.2 %	0.0264	5.1 %	0.0123	3.6 %
Log income per capita	0.0097	0.0083	−0.5136	2.8 %	3.0979	−15.6 %	−0.5525	5.2 %	0.4390	−2.1 %
Divorced/separated	0.0385	0.0254	0.0021	0.0 %	0.0171	−0.3 %	0.0069	−0.3 %	−0.0001	0.0 %
Widowed	−0.2875	−0.3389	0.0079	1.3 %	−0.0231	−4.7 %	−0.0008	−0.2 %	0.0111	2.1 %
Single/never married	−0.0358	0.0634	0.1690	3.4 %	0.2596	−9.9 %	0.0314	1.1 %	0.0095	−0.3 %
Herder	−0.3153	−0.1912	0.0124	2.2 %	0.0189	2.2 %	0.0061	1.9 %	0.0369	4.0 %
Self-employed	−0.0382	−0.0318	0.0136	0.3 %	0.1778	3.4 %	0.0061	0.2 %	0.0622	1.1 %
Inactive	−0.1876	−0.0287	0.8098	85.1 %	0.3278	5.7 %	0.1266	23.1 %	0.0548	0.9 %
Unemployed	−0.0388	0.0987	0.2135	4.6 %	0.1367	−8.1 %	0.0701	2.6 %	0.0486	−2.7 %
Lower secondary	−0.7443	−0.2015	−0.0962	−40.1 %	−0.1406	−17.1 %	−0.0524	−37.9 %	−0.0874	−10.0 %
Upper secondary	−0.0658	0.4659	−0.5781	−21.3 %	−0.3611	101.5 %	−0.2247	−14.4 %	−0.1690	44.7 %
Third-level education	0.7409	0.9270	−0.2605	108.1 %	−0.0724	40.5 %	−0.1013	73.0 %	−0.0364	19.2 %
Household size	−0.0234	0.0145	−0.3319	−4.3 %	−0.4435	3.9 %	−0.1555	−3.5 %	−0.3134	2.6 %

The contribution of each determinant (product of concentration index and elasticity of each determinant) to the EI in actual numbers is not shown in Table 4. However, the columns 5, 7, 9 and 11 display the contribution percentage of each determinant to the corresponding EI. Contribution percentage of each variable can be read as follows. For example, females aged 65 to 74 contributed 31.7 % to the measured degree of education-related inequality in chronic disease in urban areas. If females aged 65 to74 group was equally distributed across the education level, education-related inequality in chronic disease in urban areas would be 31.7 % lower. On the other hand, females aged 45 to 54 contributed -5.2 % to the degree of education-related inequality in physical limitation in urban areas. Thus, if members of this group were equally distributed across the education level, education-related inequality in physical limitation in urban areas would be 5.2 % higher.

Notably, the contributions of some determinants to the EI can exceed 100 %; nevertheless, the sum of contribution of all the determinants should be 100 %.

**Fig. 3:**
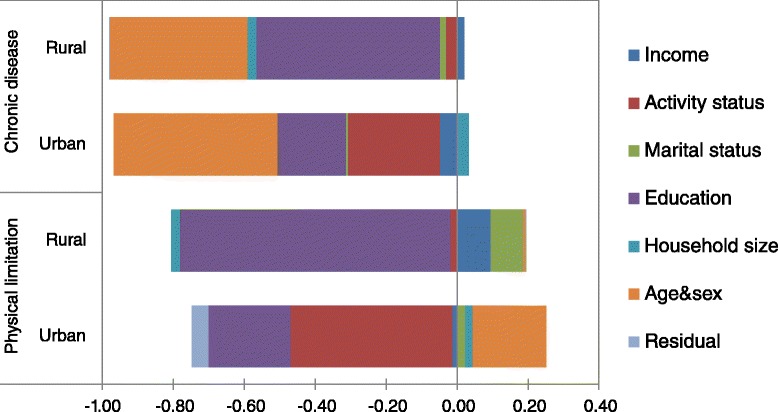
Decomposition analysis of inequalities in physical limitation and chronic disease, Mongolia, 2007/2008, by areas

Contributions of individual factors to concentration indices of education-related inequality in physical limitation and chronic disease are presented in Fig. 3.

In both areas, education-related inequality in chronic disease was mainly caused by education, economic activity status and income, except the contribution of age and sex. However, the amount education contributed to inequality was significantly larger than that of economic activity status in rural areas. The opposite was true in urban areas. The contributions of marital status and household size to inequality in chronic disease were positive but small in rural areas.

Regarding physical limitation, the main contributor was education; nevertheless, the contribution was negative in rural area. The contributions of marital status and income to inequality in physical limitation followed that of education; despite the fact that, the amount of these contributions was small. In urban areas, inequality in physical limitation was largely caused by economic activity status and education, except age and sex.

## Conclusion and discussion

In this cross-sectional study, we used the HSES 2007/2008 data to analyse and decompose education-related health inequality in Mongolia. The results revealed a number of considerable number of features.

First, we found that ill-health was unequally distributed across the population and geographic locations. Particularly, the rural population significantly suffered from chronic diseases compared to the urban population. Physical limitation was also reported to be higher in rural areas, but it was insignificant. However, health status differences among the population is a well-known characteristic in Mongolia. An early report of the Ministry of Health (MoH) documented that in 2010 the incidence of respiratory, digestive and genitourinary diseases was higher in rural areas, while there were higher incidence rates for injuries and cardiovascular diseases in urban areas [6].

Second, we used self-reported health variables as outcome variables. The findings suggested that a low education level was associated with self-reported poor health. These are consistent with an earlier study by Gan-Yadam and others; however, it was confined to the capital city, Ulaanbaatar [12].

Third, the concentration indices of physical limitation and chronic disease were significantly less than zero in both areas. It demonstrates the existence of education-related inequality in health in both urban and rural areas. On the other hand, ill-health was concentrated among the less educated group. To our knowledge, there are no other studies on education-related inequality in health in Mongolia. As mentioned earlier, one study addressed socioeconomic-related inequality in health, specifically child health, which reported that ill-health was concentrated among the lower socioeconomic groups [15].

Fourth, in terms of physical limitation, we found similar degrees of inequalities in both areas. However, when the concentration index was standardized by age and sex, a larger degree of avoidable inequality was observed in urban areas. Education-related inequality in chronic disease was significantly larger in rural areas than urban areas. However, after age-sex standardization, the difference became smaller.

The decomposition results showed that education, economic activity status and income were the main contributors to education-related inequalities in physical limitation and chronic disease, after removal of the contributions of age and sex, which similar to other results were found in both developing and developed countries [27–29].

Fifth, it is worth noting that the contribution of education to inequalities was considerably larger than the contribution of economic activity status in rural areas, while economic activity status contributed more than education to inequalities in urban areas.

It is possible that education and healthy lifestyle had more impact on rural population health. For instance, the WHO Steps survey (in 2009) indicated that 92.3 % of Mongolian population consume fruits and vegetables under the recommended intake [30]. Interestingly, the fruit and vegetable consumption of the rural population is only half of that consumed by city dwellers. They use more salt than urban people. Moreover, there was a difference in health knowledge, attitude and practice between the people living in rural and urban areas. According to study results of Alessandro R Demaio, the rural population have less knowledge about diabetes and hypertension in Mongolia [31, 32].

Social determinants of health including gender, education, occupation, and place of residence and other health system factors also could potentially contribute to the difference in health inequality between rural and urban areas [33]; since they can interact with each other [2]. Goddard and Smith stated that disparities in health care access are supply side issues and they depend on availability, quality, cost of health care services and information [34]. The government of Mongolia has been paying attention to more equitable and affordable health care services [35]. The New Health Act revised in 2011 reorganized all health care service providers according to their functions and structures at different levels. Specifically, focus of primary health care has been shifted from the former curative services to public health interventions [6]. Currently, everyone has free access to publicly funded and provided primary health care. Nonetheless, there is evidence of difference in the availability of health care services between urban and rural areas. For example, the health workforce, in general, is unequally distributed across the country. MoH data shows 21.9 doctors and 32.7 nurses per 10,000 population in rural areas, and 40.8 doctors and 37.5 nurses per 10,000 population in urban areas, in 2013 [11]. All tertiary level hospitals are located in Ulaanbaatar. Consequently, rural people, especially the lower socioeconomic groups, prefer primary health care to secondary and tertiary level care. It is not only an issue of geographical barriers and a lack of health care availability; it is also about financial barriers. A considerable proportion, 98.6%, of the total population have social health insurance coverage regardless of their socioeconomic characteristics. Out-of-pocket payment had risen to 41% of the total health expenditure in Mongolia in 2010 [6]. Moreover, a previous study concluded that 85% of inpatients consume meals prepared at home every day and about 40% of them buy drugs and injections by themselves at secondary level hospitals [36]. This situation puts a heavier burden on the lower socioeconomic groups and rural population. Dorjdagva et al. found that degrees of inequities in health care utilization in Mongolia have increased between 2007/2008 and 2012. However, the study did not make any geographical comparison [14].

The findings provide important policy implications. First, improving accessibility and quality of education, especially for the lower SES groups may reduce socioeconomic-related inequality in health in both rural and urban areas. In rural areas, there is a lack of availability and accessibility of health care services. Interventions which improve rural population education may be a more cost-effective tool than increasing health expenditures. In other words, the intervention may increase population health and income simultaneously in the long term. Second, one of the main duties of family health centres is health prevention and promotion services, which is not separable from health education. Mongolia had above 97% literacy rate for people aged 15 and above in 2010 [5]; however, health education is remarkably low among the population. Therefore, we emphasize the need to evaluate how health education is provided at the primary health care level to the population within the framework of health prevention and promotion services.

We used a well-known and robust method, the EI, to analyse education-related inequality in health. The EI was initiated to solve a drawback of the standard concentration index. We used education as a measurement of living standard.

In our previous study, we attempted to measure socioeconomic-related inequalities in health care utilization using income as a measurement of SES [14]. Income was an appropriate measurement of SES in that case, and we did not compare urban and rural areas. However, income is not a proper measurement when it comes to the comparison of urban and rural areas, at least concerning the HSES 2007/2008 data. In rural areas, where farming and the informal sector are highly prevalent and life is mainly sustained by self-production, income may not be precisely estimated. On the other hand, populations in both areas have similarly high access to education. Statistically, the gap between rural and urban areas in gross enrolment ratio regarding secondary school education has been shrinking since 2000, and access to secondary school in rural areas is only 2 % less than that in urban areas in 2011. Access to post-primary education in the country has become more equitable than it was during the 1990s [5]. Thus, this characteristic suggests that education is a better measure of SES for a comparison between rural and urban areas in Mongolia. Furthermore, evidence has shown that individual education determines future occupation and income level [37]. One additional year of education was associated with decreased mortality and increased earnings, both by 8%. This association supports the concept that education ameliorates health both in direct and indirect ways, since income also increases health independently [1].

Our findings show socioeconomic-related inequality in adult population health. Earlier studies investigated in Mongolia produced a partial picture of inequality, specifically, inequality in child health. We believe this study includes a larger scope of socioeconomic-related inequalities in health.

This study has a few limitations. The survey questionnaire did not contain any questions about respondents’ behavioural risk factors or lifestyle such as physical activity, smoking and alcohol, consumption, etc. Moreover, decomposition analysis does not allow causal interpretation.

## Abbreviations

EI: Erreygers concentration index
HSES: Mongolian household socio-economic survey
ISCED: International standard classification of education
MoH: ministry of health
OECD: The Organization for economic cooperation and development
OLS: ordinary least square
SES: socioeconomic status
WHO: World health organization

## Footnotes

1 The following are the desirable four properties of the rank-dependent indices: “i) transfer – a small transfer of health from a richer (poorer) to a poorer (richer) individual translates into a pro-poor (pro-rich) change in the index; ii) mirror – the inequality indices of health and ill-health are mirror images of each other; that is, *I*(*h*) is equal to the absolute value of *I*(1 − *h*), but has the opposite sign; iii) level independence - an equal increment of health for all individuals does not affect the index; that is, the index is invariant to scalar addition even when the bounds of the variable are kept constant; iv) cardinal invariance – a linear transformation of the health variable, *h*_*i*_, does not affect the value of the index; that is, the measured degree of inequalities is the same, irrespective of the cardinal scale of the health variable (e.g. *I*(*h*) of body temperature would be the same whether measured in Celsius or Fahrenheit)” [24].
